# Bariatric Surgery Affects Plasma Levels of Alanine Aminotransferase Independent of Weight Loss: A Registry-Based Study

**DOI:** 10.3390/jcm10122724

**Published:** 2021-06-20

**Authors:** Shira Azulai, Ronit Grinbaum, Nahum Beglaibter, Shai Meron Eldar, Moshe Rubin, Shai Carmi, Rachel Ben-Haroush Schyr, Orly Romano-Zelekha, Danny Ben-Zvi

**Affiliations:** 1 Department of Developmental Biology and Cancer Research, Institute for Medical Research Israel-Canada, The Hebrew University of Jerusalem-Hadassah Medical School, Jerusalem 91120, Israel; shira.azulai@gmail.com (S.A.); Rachel.schyr@mail.huji.ac.il (R.B.-H.S.); 2 Department of Surgery, Hadassah-Hebrew University Medical Center, Mount Scopus, Jerusalem 91240, Israel; ronitgr@hadassah.org.il (R.G.); bnahum@hadassah.org.il (N.B.); 3 Bariatric Surgery Unit, Division of General Surgery, Tel Aviv Sourasky Medical Center, Tel-Aviv 64239, Israel; shaime@tlvmc.gov.il; 4 Israel Sackler Faculty of Medicine, Tel-Aviv University, Tel-Aviv 69978, Israel; moshe.rubin2@sheba.health.gov.il; 5 Department of Surgery, Tel HaShomer Medical Center, Ramat Gan 52621, Israel; 6 Braun School of Public Health and Community Medicine, The Hebrew University of Jerusalem-Hadassah Medical School, Jerusalem 91120, Israel; shai.carmi@mail.huji.ac.il; 7 Israel Center for Disease Control, Ministry of Health, Ramat Gan 52621, Israel; orly.romano@moh.gov.il

**Keywords:** bariatric surgery, A1C, ALT, AST, registry, obesity, OAGB, RYGB, sleeve gastrectomy

## Abstract

Patients that undergo bariatric surgery experience weight loss and a reduction in the plasma levels of the hepatic enzymes alanine aminotransferase (ALT) and aspartate aminotransferase (AST). We used the Israeli national bariatric registry, which includes demographic, clinical, and biochemical data on 19,403 patients, of which 1335 patients had two-year follow-up data on ALT, AST, A1C, and BMI, to test the dependence of the reduction in the levels of ALT and AST on weight loss. The data were analyzed using regression models, retrospective matching, and time course analyses. Changes in liver enzymes did not correlate with change in BMI, and linear regression models did not demonstrate that the change in ALT and AST values were dependent on pre-operative levels of BMI or the extent of weight loss. ALT and AST levels were reduced two years after surgery compared with a cohort of retrospectively matched patients for ethnicity, sex, age, BMI, and A1C. Finally, patients who regained weight displayed a reduction in levels of liver enzymes. Our results suggest that bariatric surgery affects AST and ALT levels via weight loss dependent and independent mechanisms. Mechanistic studies that will identify the nature of this effect and the clinical relevance of ALT and AST levels to the post-bariatric liver function are warranted.

## 1. Introduction

Bariatric surgeries lead to weight loss and improvement in obesity-associated comorbidities, such as type 2 diabetes (T2D) [1,2,3,4]. Clinical and rodent studies show that the post-surgical anatomy may improve glycemic control and increase satiety through several mechanisms independent of weight loss. Glucagon-like peptide 1 (Glp1) secretion is increased following bariatric surgery, amplifying Glp1 signaling of insulin secretion from pancreatic beta cells and inducing satiety via hypothalamic signaling to POMC neurons [5,6,7,8]. The plasma levels of bile acids increase following bariatric surgeries, and bile acid signaling via the nuclear receptor FXR has been shown to mediate some of the glycemic effects of surgery [9,10,11,12]. Bariatric surgery alters the composition of the gut microbiome [13,14]. The new microbial communities affect secondary bile acid metabolism, and were shown in rodent studies to be important for weight loss [15,16]. Roux-en-Y gastric bypass (RYGB) increases intestinal basolateral glucose uptake, which can explain in part improved glycemia [17,18]. We and others have shown that sleeve gastrectomy (SG) and RYGB are associated with activation of the hepatic PPARA pathway [19,20,21,22]. These studies support the conclusion that bariatric surgery affects metabolism through numerous non-overlapping pathways.

Obesity is associated with non-alcoholic fatty liver disease (NAFLD), and it has been shown that weight loss of 10% can improve NAFLD in many cases [23,24,25]. Bariatric surgeries, which usually lead to a much greater weight loss, were shown to reduce the plasma levels of the liver enzymes alanine aminotransferase (ALT) and aspartate aminotransferase (AST) at both two and ten years after surgery in the Swedish Obesity Study [26]. Hepatic imaging and histological analysis of liver biopsies demonstrated an improvement in NAFLD and nonalcoholic steatohepatitis (NASH) without worsening of fibrosis up to five years after surgery. The degree of improvement was associated with the amount of weight loss [26,27,28,29,30,31,32,33,34]. 

Molecular studies show that hepatic DNA methylation, gene expression, and several signaling and metabolic pathways are affected by bariatric surgery, also independent of weight loss. These studies indicate that the post-bariatric liver assumes a new molecular state that is different than the state of the healthy liver or of a liver affected by NAFLD or NASH [20,21,22,35,36]. The molecular findings can be attributed to the widespread changes in endocrine signaling, bile circulation, and the new composition of the gut microbiome that follow bariatric surgery [9,37,38,39]. 

The mechanistic studies and clinical outcomes indicate that bariatric surgery has weight loss independent effects on the liver. In this study, we sought evidence supporting this claim through analysis of the Israeli Bariatric Registry [40,41,42].

## 2. Materials and Methods

### 2.1. Database

We analyzed data from the Israeli Bariatric Surgery registry from January 2014 to December 2018. The variables we used were sex, ethnicity (Jewish, Arab), surgery type (SG, RYGB, OAGB (one anastomosis gastric bypass)), pre-operative smoking (self-reported), alcohol consumption (self-reported), and diagnosis of hypertension. Numerical values were age, BMI (body mass index), glycated hemoglobin (A1C) percentage, plasma triglycerides (TG) levels, and levels of ALT and AST. Pre- and two-year post-surgery data of BMI, A1C, ALT, and AST were available from 1335 patients. Data on revision surgery were excluded. Data on alcohol consumption during the follow-up period were not available.

Abnormal ALT levels were defined as higher than 25 IU/L for females and higher than 33 IU/L for males, according to the American College of Gastroenterology [43]. Weight regain was defined as an increase in at least 25% of excess weight between one and two years after surgery [44].

### 2.2. Statistical Analysis

Continuous variables were reported as the median and interquartile range. The Mann–Whitney U test or Kruskal–Wallis H tests, followed by a post hoc Mann–Whitney U test, were used for continuous variables. The χ^2^ test was used for categorical variables. Bonferroni correction was used to correct for multiple hypothesis testing in each table or figure.

Regression models: Backward variable elimination linear regression models were used in multivariate analyses. We incorporated demographic characteristics, age, surgery type, pre-surgical ALT or AST levels, TG, BMI, and A1C, or the difference in BMI and A1C, to estimate the change in ALT or AST levels two years after surgery. Pre-surgical levels of AST and TG were not included in the models for the change in ALT, and ALT and TG were not included in the model for AST due to the high correlation between ALT, AST, and TG levels.

Matching: Pairs were matched within the same categorical subgroups of sex and ethnicity. Matched populations were obtained by choosing pairs with the minimal Euclidean distance in age, BMI, and A1C, ensuring similarity in each continuous variable included in the matching algorithm. 

All data were analyzed using scikit-learn (https://scikit-learn.org), statsmodels (www.statsmodels.org), and SciPy packages (www.scipy.org/scipylib) [45,46,47].

## 3. Results

### 3.1. BMI, A1C, ALT, and AST Levels Were Reduced Two Years after Bariatric Surgery

There was a significant reduction in the BMI, A1C, ALT, and AST of the patients two years after bariatric surgery. The median change in BMI was 11.56 kg/m^2^, and A1C was reduced by 0.8%. ALT and AST levels were reduced by 7 IU/L and 4 IU/L, respectively. The fraction of patients with abnormal ALT levels [43] was reduced from 42% to 13% (Table 1). The data stratified by surgery type are shown in Table S1. Patients for which two-year follow-up data were available had similar pre-surgical levels of BMI, ALT, and AST to the general population in the registry, but were older with a higher A1C percentage, and a larger fraction had hypertension (Table S2).

### 3.2. Changes in ALT and AST Were Not Correlated with Changes in BMI Two Years after Surgery 

**Figure 1:**
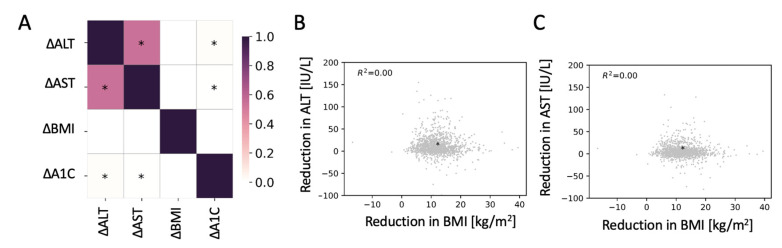
No correlation between changes in body mass index (BMI) with changes in the levels of alanine aminotransferase (ALT) or aspartate aminotransferase (AST). (**A**) Pearson correlation matrix for the changes in ALT, AST, BMI, and glycated hemoglobin (A1C) two years after bariatric surgery. * Significant correlation (*p* < 0.05). (**B**,**C**) Scatter plot of the reduction in ALT (**B**) and AST (**C**) as a function of the reduction in BMI. * Median change.

There was no correlation between changes in the levels of ALT or AST and changes in BMI two years after surgery (Figure 1A–C). A very small but significant positive correlation between the reduction in A1C and ALT or AST was detected. The change in levels of ALT and AST were highly correlated.

### 3.3. Pre-Surgical Levels of Liver Enzymes, Age, and Surgery Type Contributed to a Regression Model for Changes in the Levels of ALT or AST Two Years after Surgery

Pre-surgical levels of BMI or A1C did not contribute to a multivariate linear regression model for the change in the levels of ALT after surgery. Replacing pre-surgical levels with changes in BMI and A1C two years after surgery did not contribute to the model. Rather, high pre-surgical ALT, young age, and having SG and not RYGB or OAGB predicted a larger reduction in ALT levels (Table 2). Only pre-surgical ALT levels and surgery type contributed to a model on the subpopulation of patients with abnormal pre-surgical ALT levels [43] (Table 3). Likewise, pre-surgical AST levels, age, and surgery type contributed to a model for the post-surgical change in AST levels (Table S3).

### 3.4. Retrospective Matching of Pre- and Post-Surgery Patients Showed a Reduction in ALT and AST Levels Following Bariatric Surgery

**Figure 2:**
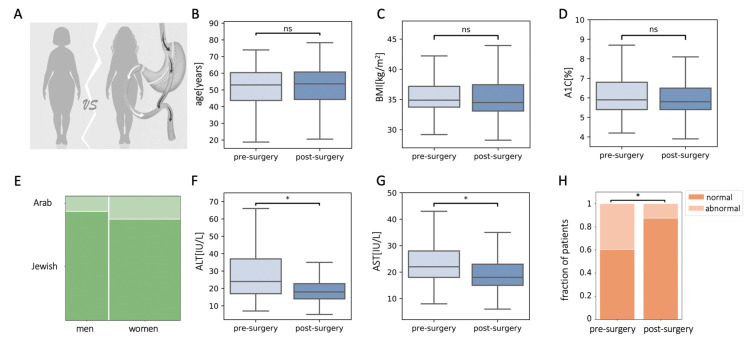
Plasma levels of alanine aminotransferase (ALT) and aspartate aminotransferase (AST) in patients that underwent bariatric surgery were reduced compared to retrospectively matched pre-operated patients. (**A**–**E**) Characteristics of matched populations: age (**B**), body mass index (BMI) (**C**), glycated hemoglobin (A1C) (**D**), and sex and ethnicity (**E**). (**F**,**G**) Levels of ALT (**F**) and AST (**G**) were lower in the matched post-surgery population. (**H**) The fraction of patients with abnormal ALT levels were reduced in the matched post-surgical population; ns, non-significant, * *p* < 0.01 by Mann–Whitney U test (**C**,**D**,**F**,**G**) or χ^2^ test (**H**) and Bonferroni correction.

We retrospectively matched 446 pre-operative patients from the registry, with 446 post-operative patients from our study cohort for age, sex, ethnicity, BMI, and A1C (Figure 2A–E). ALT and AST levels of patients two years post-surgery were lower than that of matched pre-surgery patients (Figure 2F,G). There were 177 patients (40%) who had abnormal ALT levels in the pre-surgical population, and only 56 patients (13%) with abnormal ALT levels in the matched post-surgical population (Figure 2H).

### 3.5. Patients Who Regained Lost Weight Maintained Low ALT and AST Levels Two Years after Surgery

**Figure 3:**
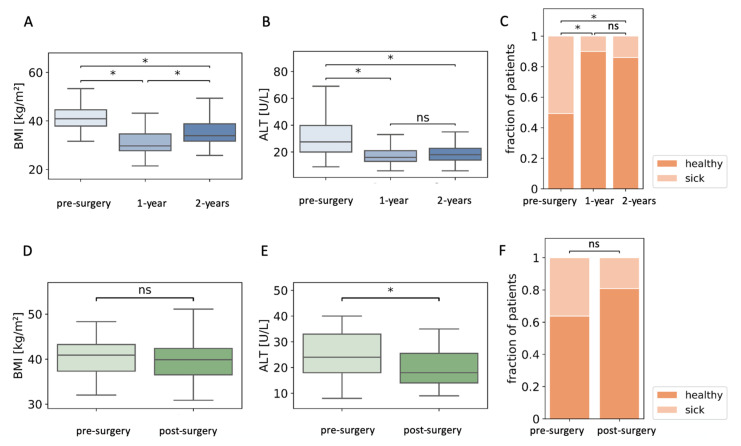
Alanine aminotransferase (ALT) levels are reduced in patients who regain lost weight. (**A**) Body mass index (BMI) of patients who regained at least 25% excess weight loss during the second year after surgery. (**B**) ALT levels in the same group of patients. (**C**) Fraction of patients with abnormal levels of ALT pre-surgery, and one and two years after surgery. (**D**) BMI of patients that did not lose weight two years after surgery. (**E**) Pre- and two-year post-surgery ALT levels of the same group of patients. (**F**) The fraction of patients that did not lose weight that had abnormal ALT levels before surgery and two years after surgery; *n* = 150 (**A**–**C**); *n* = 47 (**D**–**F**); ns, non-significant; * *p* < 0.05 by Kruskal–Wallis and Mann–Whitney post-hoc U test (**A**,**B**), Mann–Whitney U test (**D**,**E**), or χ^2^ test (**C**,**F**), followed by Bonferroni correction for all tests.

We identified patients in the dataset who had follow-up data on levels of ALT and BMI at one- and two-years post-surgery, and who regained over 25% excess weight loss (*n* = 150, Figure 3A) [44]. ALT levels remained low in the second year despite weight regain (Figure 3B). Seventy-six of these 150 patients had abnormal ALT levels before surgery, and 15 patients retained abnormal ALT levels one year after surgery. Relapse of high ALT levels concomitant with weight regain occurred in only six patients (Figure 3C). AST levels were reduced significantly (*p* < 0.05) from 26 (20–34) IU/L before surgery to 19 (16–23) IU/L one year after surgery. AST levels did not change after partial regain two years after surgery (21 (17–23.0) IU/L).

We identified 47 patients who did not lose weight two years after surgery (∆BMI: 0.87 (0.35–1.49) kg/m^2^). The final levels of ALT in these patients were significantly lower two years after surgery. In particular, 17 of the 47 patients had abnormally high ALT levels before surgery, while only nine had abnormally high levels two years later (Figure 2D–F). AST levels did not change significantly in these patients: 21.0 (19.0–32.3) IU/L pre-surgery and 20.50 (16.3–24.0) IU/L two years after surgery.

## 4. Discussion

Bariatric surgery often causes weight loss and a reduction in the levels of A1C, ALT, and AST in patients with obesity [3,26,27,28,29,30,31,32,48,49,50]. We analyzed two-year follow-up data from the Israeli Bariatric Registry and provided evidence that the reduction in the levels of liver enzymes is not dependent on weight loss or reduction in A1C, although weight loss and reduction in A1C, ALT, and AST levels occur simultaneously. The population we analyzed is older and has higher A1C levels than the general population in the registry (Table S2). This bias may have contributed to our ability to identify an effect of age on the reduction in ALT levels, and increases the confidence that the effect of A1C on reduction in liver enzymes is not major in this dataset. 

There was no correlation between the changes in ALT or AST and changes in BMI. This is unexpected, considering previous studies. For example, Burza et al. analyzed the Swedish Obesity Study database [26] and have noted such correlations. The variations in the findings can be attributed to differences in study populations. For example, in our study, SG was the predominant surgery, and Jewish and Arab ethnicities are unique to this study. There are also differences in local diet [51], which is mostly Mediterranean in adherence to dietary recommendations [52], and in post-operative healthcare in different countries. 

We constructed a multivariate regression model to gain better insight into the role of changes in BMI and A1C in alterations in the levels of plasma liver enzymes. This model includes demographic parameters, surgery type, preoperative comorbidities, smoking and alcohol consumption, ALT or AST levels, A1C or change in A1C levels, and BMI or change in BMI two years after surgery. Neither change in BMI and A1C nor pre-surgical levels of BMI and A1C were associated with the change in ALT or AST levels. SG surgery type, younger age, and high pre-surgical levels of ALT or AST significantly contributed to a greater reduction in liver enzymes. These results also apply to patients who had abnormal pre-surgical ALT levels. There is a complex relationship between age and outcomes of bariatric surgeries. Younger age was associated with a higher risk for weight regain [44,53] and a lower risk for diabetes remission after surgery [54]. In our models, older age was associated with a smaller reduction in liver enzymes. 

High ALT levels are associated with hyperglycemia, insulin resistance, and the risk of developing T2D [55]. We found a very low yet significant correlation between changes in ALT or AST levels and changes in A1C percentage. While changes in A1C and ALT were correlated, the change in A1C did not contribute to the linear regression model. Previous studies have shown that the fraction of patients that use T2D drugs decrease following bariatric surgery [56], which makes the interpretation of changes in A1C levels challenging. Data on the number and types of medications used by patients before and after surgery were not available to us in this study. Therefore, we cannot exclude that improvement in glycemia after bariatric surgery plays an important role in the reduction in ALT levels. 

The finding that SG contributed to a greater reduction in ALT in the regression model despite lesser or equal weight loss and reduction in A1C compared with bypass surgeries supports the hypothesis that factors related to bariatric surgery [37,57] and not only to weight loss affect the levels of liver enzymes. In particular, SG, which leads to lesser or equal weight loss or reduction in A1C, leads to a greater reduction in AST and ALT levels [3,50,58,59,60,61,62,63] (Table S1). 

To test whether a history of surgery affects ALT or AST levels, we matched BMI, A1C, and demographic parameters between patients before surgery and patients two years after surgery. The post-operative patients had lower levels of AST and ALT, and the fraction of patients with abnormal ALT levels was three-fold smaller: 40% of the pre-operative patients had abnormal ALT levels, and 13% of the matched post-operative patients had abnormal ALT levels. Therefore, having bariatric surgery can reduce the occurrence of abnormal ALT levels by 67%. This finding supports the impression that bariatric surgery has an effect on liver enzymes. It is important to note that the pre-operative patients had obesity, but were leaner and had lower glycemia than most patients that undergo surgery, while the post-operative patients had obesity after surgery. Moreover, the post-operative patients had already experienced weight loss, and therefore, it is possible that significant weight loss, rather than current BMI, is the important factor in reducing ALT and AST levels after bariatric surgery [23,24,25]. 

If indeed a reduction in ALT and AST is mostly a result of weight loss, then we would expect that weight regain [44] would induce an increase in AST and ALT levels. However, when we tested AST and ALT levels of 150 patients who displayed a 25% regain of excess weight loss between the first and second year after surgery, we did not detect a significant increase in ALT or AST. Seventy-six of those 150 patients had abnormal pre-operative levels of ALT; 80% (61/76) normalized their ALT levels one year after surgery. Even though all of these patients experienced at least 25% regain of excess weight loss, only 10% (6/61) developed abnormal ALT levels in the following year. Similarly, AST levels were reduced in this group of patients one year after surgery, and remained low despite regain. Studies using other definitions of weight regain [44] had similar qualitative results.

We identified 47 patients with obesity who did not display any weight loss two years after bariatric surgery, likely due to a complete regain of weight. Even in this population, the median ALT level was significantly lower two years after surgery, and 47% (8/17) of the patients who had abnormal ALT levels before surgery normalized their ALT levels with no weight loss. It will be important to study whether the effect of bariatric surgery on ALT levels after weight regain is temporary. AST levels did not change in this group of patients, yet AST levels were not high pre-operatively. Altogether, our results provide evidence that factors induced by bariatric surgery, other than weight loss and improvement in glycemia, contribute to the reduction in the plasma levels of ALT and AST. 

Notably, ALT levels were reduced to their post-surgical levels just six months after surgery, even in patients with high pre-surgical ALT. Weight loss is usually a longer process, and the reduction in BMI six months after surgery was not reflected in changes in ALT levels, supporting a lack of correlation between changes in weight one year after surgery and changes in ALT (Figure S1).

In a recent clinical study, it was shown that RYGB has a greater effect on the histological NAFLD activity score (NAS) than gastric banding five years after surgery, and that this effect cannot be explained by weight loss alone [34]. In the same study, an average increase in BMI of 2.5 kg/m^2^ from weight regain that was experienced by patients between one and five years after RYGB surgery did not affect NAS score [34]. In another study, 60% of the patients that experienced a relatively small weight loss of less than 5 kg/m^2^ five years after bariatric surgery experienced resolution of NASH without worsening of fibrosis according to histological scoring [28]. Fibrosis improved and NASH did not progress between one and five years after surgery, even though some degree of weight regain is common in that period [28]. A small reduction in weight following bariatric surgery leads to a large improvement in NASH in the long term, and this may be reflected in the sustained reduction in the levels of liver enzymes we show here. 

Several mechanisms may be responsible for driving a reduction in the levels of ALT and AST following bariatric surgery. The level of circulating bile acids is increased after bariatric surgery and their composition is modified, possibly following a change in the gut microbiome. Upregulation in bile acid signaling in the liver may underlie some of the weight loss independent effects of surgery [9,10,12]. There is accumulating evidence that the gut microbiome contributes to the pathogenesis of NAFLD, and significant shifts in the composition of the gut microbiome were found following bariatric surgery [13,14,64]. It remains to be tested whether these shifts in the gut microbiota following bariatric surgery affect liver function. PPARA agonists were shown to reduce the levels of liver enzymes in clinical studies [65], and PPARA signaling is increased in the liver following bariatric surgery dependent and independent of weight loss [19,20,21,22], suggesting another mechanism for reduction in ALT levels. 

Histological studies show that the extent of improvement in NAS score following bariatric surgeries in patients with NAFLD is greater in patients that experienced a larger weight loss [26,27,28,29,30,31,32,33,34]. A significant weight regain was associated with worse liver histology three years after RYGB [33]. It is possible that the reduction in the plasma levels of liver enzymes after bariatric surgery does not correlate well with histological findings [63], especially in longer follow-up studies. This interpretation has an important implication, since ALT and AST levels are used routinely as biomarkers for liver injury in patients that had bariatric surgery. Notably, PPARA agonists were shown to reduce the levels of ALT in several studies without affecting hepatic histology [65].

An important limitation of our study is that we do not have information on the status of NAFLD before or after surgery. Furthermore, we do not have information on the physical activity, dietary habits, medications, or levels of insulin and bilirubin before and after surgery. Alcohol consumption is self-reported only before surgery. All of these factors may affect the outcome of bariatric surgery and levels of liver enzymes [66,67,68]. A second limitation is that our analysis is based on BMI, which gives only partial information on the obesity of the patients. Other parameters, such as hip and waist circumference, lean mass, and fat mass, may be more indicative of obesity and improvement in obesity following bariatric surgery [69,70]. Another limitation of our study is the short follow-up period. A period of two years is close to the nadir of weight loss and reduction in A1C. A future study with a longer follow-up is warranted. 

## 5. Conclusions

This two-year study based on data from the Israeli Bariatric Surgery registry suggests that a reduction in AST and ALT levels following bariatric surgery is in part independent of weight loss and reduction in A1C levels. Mechanistic studies [9,19,20,22] are needed to identify how surgery can impact the levels of liver enzymes. Clinical studies using imaging modalities and pathological examinations are required to assess the clinical significance of AST and ALT levels in particular after bariatric surgery. Table S1 shows the mean ALT and BMI values six, 12, and 24 months after surgery of each decile in the study population according to the patients’ pre-operative values.

## Figures and Tables

**Table 1:** Characteristics of patients in the registry with two-year follow-up data.

	Population Characteristics Pre-Surgery	Population Characteristics Post-Surgery
ALT (IU/L) *	26 (19–38)	18 (14–23)
Patients with abnormal ALT levels *	560 (42%)	176 (13%)
AST (IU/L) *	23 (18–30)	19 (15–23)
A1C (%) *	6.5 (5.9–7.7)	5.6 (5.3–6)
BMI (kg/m^2^)*	41.21 (38.39–44.55)	29.62 (26.51–32.90)
EWL (%)	71.69 (54.80–90.33)	---
TG (mg/dL)	156 (114–216)	---
Age (years)	51.7 (41.9–58.9)	---
Female sex	830 (62%)	---
Jewish ethnicity	1138 (85%)	---
Smoking	217 (16%)	---
Hypertension	699 (52%)	---
Alcohol consumption	25 (2%)	---
Surgery type	SG: 1073 (80%) RYGB 168 (13%) OAGB 94 (7%)	---

Median and interquartile ranges are shown; * *p* < 0.01 between pre- and post-surgery parameter using the Mann–Whitney U test and Bonferroni correction. ALT: alanine aminotransferase; AST: aspartate aminotransferase; BMI: body mass index; A1C: glycated hemoglobin; TG: triglycerides; SG: sleeve gastrectomy; RYGB: Roux-en-Y gastric bypass; OAGB: one anastomosis gastric bypass.

**Table 2:** Backwards elimination multivariate linear regression model for the change in ALT levels two years after bariatric surgery.

	β	SE β	*p*-Value	CI
Constant	−11.31	1.65	<1 × 10^−5^	(−14.56, −8.01)
ALT (IU/L)	0.89	0.02	<1 × 10^−5^	(0.85, 0.92)
Age (years)	−0.08	0.03	<0.001	(−0.14, −0.02)
RYGB vs. SG	−5.67	1.07	<1 × 10^−5^	(−7.77, −3.58)
OAGB vs. SG	−5.94	1.38	<1 × 10^−5^	(−8.65, −3.24)

β: regression coefficient; SE: standard error; CI: 95% confidence interval; ALT: alanine aminotransferase; SG: sleeve gastrectomy; RYGB: Roux-en-Y gastric bypass; OAGB: one anastomosis gastric bypass. *n* = 1355, R^2^ = 0.66.

**Table 3:** Backwards elimination multivariate linear regression model for the change in ALT levels two years after bariatric surgery in patients with abnormal pre-surgical ALT levels.

	β	SE β	*p*-Value	CI
Constant	−16.06	1.22	<1 × 10^−5^	(−18.46, −13.65)
ALT (IU/L)	0.91	0.02	<1 × 10^−5^	(0.86, 0.95)
RYGB vs. SG	−6.20	1.59	<1 × 10^−5^	(−9.31, −3.09)
OAGB vs. SG	−4.89	2.07	<0.05	(−8.95, −0.83)

β: regression coefficient; SE standard error; CI: 95% confidence interval; ALT: alanine aminotransferase; SG: sleeve gastrectomy; RYGB: Roux-en-Y gastric bypass; OAGB: one anastomosis gastric bypass. *n* = 560, R^2^ = 0.74.

## Data Availability

The data presented in this study are available on request of the corresponding author, due to the request of the institutional review board.

## Supplementary Materials

The following are available online at https://www.mdpi.com/article/10.3390/jcm10122724/s1. Table S1: Characteristics of patients in this study stratified by bariatric surgery type. Table S2: Pre-surgical characteristics of all patients in the registry and of the patients for whom two-year follow-up data on BMI, A1C, AST, and ALT were available. Table S3: Parameters for backwards elimination multivariate linear regression model for the change in AST levels two years after bariatric surgery.
