# Supplementary material for: Bariatric Surgery Affects Plasma Levels of Alanine Aminotransferase Independent of Weight Loss: A Registry-Based Study
Source: J Clin Med. 2021 Jun 20;10(12):2724. doi: 10.3390/jcm10122724 (PMC8234536; doi:10.3390/jcm10122724)
Supplement: Supplementary file 1 [file jcm-10-02724-s001.zip › jcm-1202159-supplementary.pdf]

# Bariatric surgery affects plasma levels of alanine aminotransferase independent of weight-loss: a registry-based study

**Shira Azulai [1], Ronit Grinbaum [2], Nahum Begblaibter [2], Shai Meron Eldar [3,4], Moshe Rubin [4,5], Shai Carmi [6], Rachel Ben-Haroush Schyr [1], Orly Romano-Zelekha [7] and Danny Ben-Zvi [1,*].**

1 Dept. of Developmental Biology and Cancer Research, Institute for Medical Research Israel-Canada, The Hebrew University of Jerusalem-Hadassah medical school, Jerusalem, Israel; shira.azulai@gmail.com; Rachel.schyr@mail.huji.ac.il
2 Dept. of Surgery, Hadassah-Hebrew University Medical Center, Mount Scopus, Jerusalem, Israel; ronitgr@hadassah.org.il; bnahum@hadassah.org.il
3 Bariatric Surgery Unit, Division of General Surgery, Tel Aviv Sourasky Medical Center, Tel-Aviv, Israel; shaieldar@gmail.com
4 Israel Sackler Faculty of Medicine, Tel-Aviv University, Tel-Aviv, Israel
5 Dept. of Surgery, Tel HaShomer Medical Center, Ramat Gan, Israel, Israel; moshe.rubin2@sheba.health.gov.il
6 Braun School of Public Health and Community Medicine, The Hebrew University of Jerusalem-Hadassah medical school, Jerusalem, Israel; shai.carmi@mail.huji.ac.il

7 Israel Center for Disease Control, Ministry of Health, Ramat Gan, Israel; orly.romano@moh.gov.il
* Correspondence: danny.ben-zvi@mail.huji.ac.il

# Supplementary Information

| | SG (n=1073 ) | RYGB (n=168 ) | OAGB (n=94) |
|---|---|---|---|
| ALT pre-surgery [IU/L] | 26 [19-39] | 24 [18-36] | 25.5 [20-33.75] |
| ALT post-surgery [IU/L]* | 17 [13-22] | 21 [16-28] | 21.5 [17-28] |
| Patients with abnormal ALT levels pre-surgery | 459 (43%) | 65 (39%) | 36 (38%) |
| Patients with abnormal ALT levels post-surgery* | 110 (10%) | 43 (26%) | 23 (24%) |
| AST pre-surgery [IU/L] | 23 [18-30] | 23 [18.75-30.25] | 23 [19-28] |
| AST post-surgery [IU/L]* | 18 [15-22] | 23 [18.75-29] | 23 [18-28] |
| A1C pre-surgery [%]* | 6.5 [5.8-7.6] | 7.2 [6.1-8.7] | 6.3 [6-7.68] |
| A1C post-surgery [%]* | 5.6 [5.3-6] | 5.7 [5.4-6.4] | 5.4 [5-5.7] |
| BMI pre-surgery [kg/m$^2$] | 41.34 [38.57-44.6] | 40.15 [37.58-43.96] | 41.2 [38.06-43.86] |
| BMI post-surgery [kg/m$^2$]* | 30.07 [26.77-33.24] | 28.33 [25.67-31.27] | 27.2 [24.92-30.12] |
| EWL [%]* | 69.81[53.2-88.04] | 77.62 [60.74-95.13] | 83.95 [69.84-100.53] |
| TG pre-surgery [mg/dL] | 157 [115-215.8] | 156.5 [114.25-221.75] | 152.75 [101.93-212] |
| Age [years]* | 51.29 [41.08-58.41] | 55.37 [46.44-60.72] | 52.67 [43.94-59.77] |
| Female sex | 672 (63%) | 101 (60%) | 57 (61%) |
| Jewish ethnicity | 909 (85%) | 144 (86%) | 85 (90%) |
| Smoking | 178 (17%) | 25 (15%) | 14 (15%) |
| Hypertension* | 539 (50%) | 108 (64%) | 52 (55%) |
| Alcohol Consumption* | 16 (1%) | 9 (0.5%) | 0 (0%) |

Table S1: pre- and two-year post-surgery outcomes for the three surgery types in the study population. BMI: body mass index; A1C: glycated hemoglobin; TG: triglycerides. SG: sleeve gastrectomy; RYGB: Roux en Y Gastric Bypass; OAGB: one anastomosis gastric bypass. p-values derived using Kruskal-Wallis H-tests for continuous variables and using $\chi 2$ test for categorical variables. Bonferroni correction was used to correct for multiple hypothesis testing. *p-value<0.05 in all three surgery types.

| | **All cohort (n=19403)** | **population characteristics (n=1335)** |
|---|---|---|
| ALT pre-surgery [IU/L] | 26 [18-38] | 26 [19-38] |
| AST pre-surgery [IU/L] | 22 [18-29] | 23 [18-30] |
| A1C pre-surgery [%]* | 5.8 [5.4-6.3] | 6.5 [5.9-7.7] |
| BMI pre-surgery [kg/m$^2$] | 41.23 [38.83-44.52] | 41.21 [38.39-44.55] |
| TG pre-surgery [mg/dL]* | 144 [106-200] | 156 [114-216] |
| Patients with abnormal ALT levels pre-surgery | 8327 (43%) | 560 (42%) |
| Hypertension* | 6057 (31%) | 699 (52%) |
| smokers | 4146 (21%) | 217 (16%) |
| Alcohol consumption | 499 (3%) | 25 (2%) |
| Age [years]* | 42.2 [32.2-51.8] | 51.7 [41.9-58.9] |
| Sex – female* | 12903 (67%) | 830 (62%) |
| Ethnicity – Jewish* | 15850 (82%) | 1138 (85%) |
| Surgery type* | SG: 14880 (77%) | 1073 (80%) |
| | RYGB: 1661 (8%) | RYGB: 168 (13%) |
| | OAGB: 2862 (15) | OAGB: 94 (7%) |

Table S2: Pre-surgery parameters of patients from the registry and from study population. ALT: alanine aminotransferase; AST: aspartate aminotransferase; BMI: body mass index; A1C: glycated hemoglobin; TG: triglycerides; SG: sleeve gastrectomy; RYGB: Roux en Y gastric bypass; OAGB: one anastomosis gastric bypass.

p-values derived using Mann-Whitney U-test for continuous variables and using $\chi^2$ test for categorical variables. * p value<0.05

| | β | SE β | p value | CI |
|---|---|---|---|---|
| **Constant** | -11.83 | 2.17 | <1e-5 | [-16.07 -7.58] |
| **AST [IU/L]** | 0.89 | 0.04 | <1e-5 | [0.82 0.95] |
| **Age [years]** | -0.1 | 0.04 | <0.001 | [-0.18 -0.03] |
| **RYGB vs SG** | -5.45 | 1.42 | <1e-5 | [-8.23 -2.67] |
| **OAGB vs SG** | -5.72 | 1.83 | <0.001 | [-9.31 -2.13] |

Table S3: Backwards elimination multivariate linear regression, predicting change in AST. β: Coefficient, SE: Standard Error, OR: Odds Ratio, CI: Confidence Interval, NA: not applicable, SG: sleeve gastrectomy. RYGB: Roux en Y gastric bypass. OAGB: one anastomosis gastric bypass. ALT: alanine aminotransferase. N=1355. $R^2$=0.34.

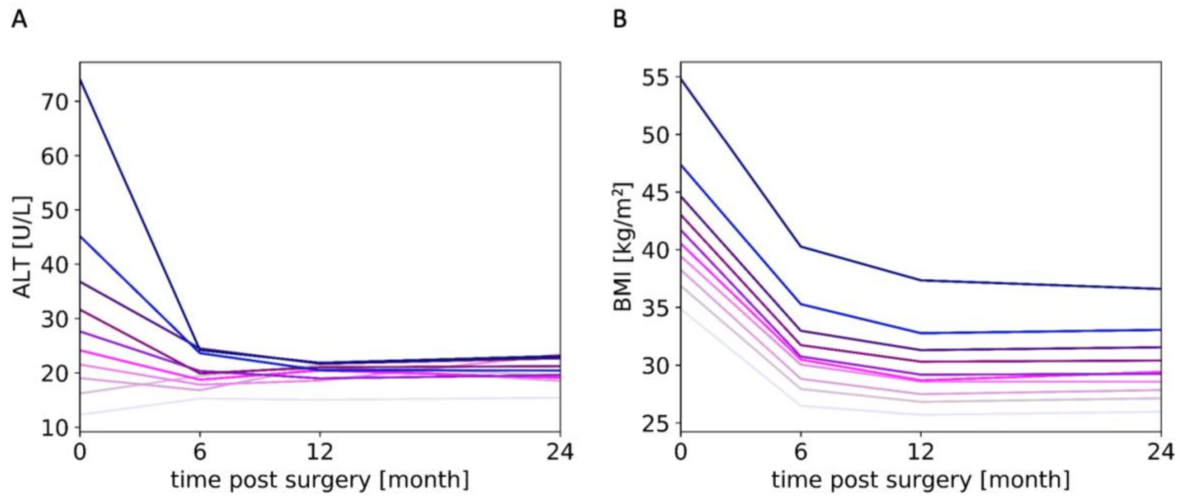


Figure S1: The mean ALT (A) and BMI (B) values of each decile in the study population where this data was available, according to the patients' pre-operative values. Highest decile in dark purple, middle decile in shades of pink and bottom decile in light purple. n=887. BMI: body mass index; ALT alanine aminotransferase.
